# Camelids: an old family spread over four continents

**DOI:** 10.1093/af/vfac045

**Published:** 2022-08-12

**Authors:** Bernard Faye, Marcelo H Ratto

**Affiliations:** UMR SELMET, CIRAD-ES, Montpellier, France; Biotechnology Department, Al-Farabi National Kazakh University, Almaty, Kazakhstan; Instituto de Ciencia Animal, Facultad de Ciencias Veterinarias, Universidad Austral de Chile, Valdivia, Chile

The large (dromedary and Bactrian camel) and small (llama and alpaca) camelids are an old family of domestic animals used by humanity for thousands of years, but until recently camelids were bred in only very specific ecosystems (desert for large ones and highlands for small ones) and in a traditional way. This close relationship between the animal and its ecosystem has maintained the camelid family in a certain marginality, in terms of demography (nowadays, the total size of camelid herd worldwide is not more than 50 million heads far less than other domestic herbivorous). Yet, for 30 to 40 years, we have observed two important changes in the camelid breeding. First in their farming system with the emergence of more intensified farming, and second, in the geographical expansion of those species, even into completely new ecosystems. It is no coincidence that the European Association of Animal Production has opened a special session on the camelid sector for their annual meeting in Western Europe.

However, those changes have several consequences for scientists. Indeed, the intensification process in camelid farming requires scientists to describe and understand the impact of those changes on the physiology, metabolism, and health of the animals, to set up and develop new biotechnologies for a more rational breeding (feeding, reproduction), to expand knowledge regarding their products (milk, meat, wool), or to investigate their biodiversity for implementing a reasoned selection policy. It is the objective of the present special issue to offer to the readers of *Animal Frontiers*, some examples of the current questions and perspectives regarding this family. With remarkable biological models as well as interesting producing animals and elements of remote ecosystems, the different questions regarding the species of this family could stimulate animal scientists to be more involved in “camelidology.”

The first paper (Faye) of this special issue, proposed by B. Faye, is asking the question of the current changes in camelid demography worldwide with a focus on large camelids. The title is formulated as a provocation (“Is the camel conquering the world?”), but as demonstrated in the paper, the camel has continued its expansion from its domestication from similar desert ecosystems in the Arabian Peninsula (for dromedary) and in Central Asia (for Bactrian). The new situation is the implementation of new camel farming systems out of their classical biotope as well in more humid ecosystems of Africa or Asia as in the temperate milieu of Europe and North America. In that new context, the camel becomes less the “ship of the desert” and more and more a productive animal for the modernized agricultural sector.

Obviously, as indicated above, the expansion of the camelid family is not only a matter of geography, but also of function. The contribution of Nagy et al. is based on the strong experience of the authors who are managing the biggest intensive camel dairy farms in the world where camels are bred “as dairy cows.” The title of the paper (Intensification of camel farming and milk production with special emphasis on animal health, welfare, and the biotechnology of reproduction) gives clearly the main elements comprising the intensification process. The authors underlined the importance of good management practices. Indeed, intensive farming systems appear *a priori* as the opposite approach to the “traditional and harmonious way of life” of the camel in their desert in terms of welfare. Yet, if the intensive management could disturb the camel metabolism oriented to water and food shortage management by providing access to a good feeding and hygienic environment, this could facilitate the safe production (milk) of “happy and healthy” camels.

Those authors have also mentioned the interest in assisted reproductive technologies to enhance the efficiency of a selective breeding program, but one constraint in the camelid family is the low reproductive performance, a limited factor of the intensification. Notably, camelids are classified as induced ovulatory species where copulation is the main stimuli for ovulation induction. The review of Paiva et al. entitled “The ovulatory and luteotropic actions of the male-derived nerve growth factor (β-NGF) in South American Camelids” gives an interesting overview of the mechanisms explaining this particularity of the members of the camelid family. The authors underline notably the role of the neurotrophin β-NGF and its potent luteizing hormone-releasing effect, leading scientists to consider that it could be possible to use this molecule as a biotechnology tool to enhance fertility in intensive farm systems. Indeed, if the induced ovulation can be regarded as an adaptation process in a context marked by harsh conditions, it might be possible to get away from this constraint in more favorable food and health contexts.

We should also highlight that interesting advance has been made to understand the genetic mechanism of fiber traits. In this sense, the paper of Anello et al. described a detailed review regarding the state of the art on genetic factors that could influence the quality of fibers of South American Camelids. Mutations have been identified as responsible for some monogenic or oligogenic traits enabling molecular testing to assist breeding decisions. The recently developed 76K single nucleotide polymorphisms array for the alpaca will facilitate the identification of genes affecting more complex traits through genome-wide association studies.

It is known that feeding is one of the important pillars of animal breeding. The camels accustomed to spending more than 8 hours a day grazing in extensive systems can be disturbed in intensive farms where they received more concentrated energetic (and proteic) feeds. Thus, the investigation of the ruminal flora could provide useful information on the microorganisms (bacteria, protozoa) and their functions to improve the feeding efficiency in such new context. The paper of Al-Jassim focused on the digestive microflora (“Foregut microbiology of the Arabian camel”) is an interesting review regarding the up-to-date knowledge of both the identity of this population and its physiological and biochemical characteristics. Moreover, the paper also emphasizes the fact that a part of the bacterial community in the camel gut is still unknown in the public database, thus opening a field of new investigations. Moreover, the co-habitation of camels and cattle in the same grazing area could lead to an alteration of this bacterial community in both species.

Historically, large camelids were animals of the nomads, merchants, and warriors. As such, they moved frequently in the arid and semi-arid parts of the world, following war and trade routes across important deserts in Africa and Asia. Consequently, the mixing of different camel “breeds” throughout history leads to a panmictic population with a certain low diversity of their genotypes. Until recently, phenotypes only were investigated to identify the ecotypes (rather than breeds as the selection process by farmers being small). It is, for example, the case of Akhmetsadykova et al. who underline the interest of these kind of studies, especially in a country like Kazakhstan where both domestic camel species (dromedary and Bactrian) are cohabiting, sometimes in the same farm. Beyond the genetic diversity, and the practice of crossbreeding between these two species, the paper recalls the cultural and economic importance of camel in the country. Milk, meat, and wool productions, even if they appear globally marginal in the country, the relative increase of the camel part seems to emphasize the role of these animals in a future marked by climatic changes.

However, in less than 20 years, camel genetics has made great progress, notably with the use of molecular tools and genomic investigation. The three following papers are devoted to camel biodiversity. Kohler-Rollefson is exploring how to conserve this biodiversity (“Camel Biodiversity—and how to conserve it”). The author is recalling that the current biodiversity is the result of farmers’ practices throughout history. Indeed, the cameleers have achieved an empirical selection to obtain phenotypes adapted to certain functions (packing, riding, milking, fattening, racing, etc.), but with the introduction of biotechnologies of reproduction in intensive systems, the risk is linked to the narrowing of gene pools. Consequently, the preservation and conservation of the biodiversity, as for other species, is to maintain in-situ breeding supported by local communities. In such context, the author concludes that such approach will have a double advantage: to ensure the conservation of camel biodiversity and to strengthen the food security of the local population living with their camels.

In their paper, the Chinese team working on Bactrian camel genomic (Liang et al.) also considers the importance of biodiversity conservation. After a nice phenotype description of the different Bactrian breeds in Central Asia (China, Kazakhstan, and Mongolia), and the current knowledge regarding the history of double-humped camel domestication, the authors of the paper “Review of genetic diversity in Bactrian camel (*Camelus bactrianus*)” write about the use of genomic tools to measure the genetic divergence between different strains of Bactrian on the Asian continent. Thus, it is assumed that Bactrian camels were first domesticated in Central Asia less than 4.45 thousand years ago, probably in more western areas than previously believed (i.e., North Iran, South Kazakhstan), and then migrated back to East Asia around 240,000 years ago. The biodiversity is then explored by using new technologies, such as mitochondrial genome, nuclear genome, and whole-genome sequencing, allowing a good understanding of the current variability. The authors also consider the importance of preserving the wild Bactrian camel which is appearing as a full-fledged species different from the domestic Bactrian.

In the paper “Structural and functional genomics in Old World camels—where do we stand and where to go” written by Burger and Ciani, the main point is the link between identified genes and different important functions in the camel, i.e., their immune responses, their environmental adaptation notably to heat stress, the genes involved in domestication process, the genes responsible of the morphological phenotypes, growth and coat color, and the genes involved in zootechnical performances as milk and meat production (quantitative trait locus, myostatin genes). For the future, the authors suggest focusing research on pangenomes and comparative genomics, (large-scale genotype-to-expression characterization, large phenotype collections, and germplasm banking). Such an approach could support a better knowledge of diversity in relation to adaptation, production traits, and performance.

Finally, to complete this special issue, it was necessary to consider camel production which was the subject of the two last papers, devoted to milk and meat, the most economically important products of large camelids (the wool being especially essential for the small camelid sector). The paper from Konuspayeva et al. focused on camel milk (“Mineral status in CM: a critical review”) is a tentative response to the common assertion that “camel milk is rich in minerals” contributing to its exceptional nutritive value. Yet, the analysis of the scientific literature indicates results conflicting with this “rich milk” narrative. Indeed, except for the cations (potassium, sodium, and chlorides) which play an important role in water metabolism and electrolytic balance, the other major minerals (calcium, phosphorus, magnesium) are not exceptionally high and even, according to some authors, in less quantity than in cow milk. It is the same for trace elements (like for vitamins except vitamin C) which are in similar or lower concentrations than cow milk, except probably iron and zinc. Beyond this description, the authors emphasize the lack of research looking for the impact of the diet composition on mineral status of camel milk

The last paper from Kadim et al. entitled “Nutritional values and health benefits of dromedary camel meat” aims to investigate the functional properties of the camel meat. As with camel milk, camel meat is regarded as a healthful product for regular consumers. It is known that the protein quality (in terms of amino acid composition) of camel meat is the highest among all common meat consumed by humans. It should also be recalled that camel milk is characterized by a low concentration of cholesterol and globally fat proportion. But the authors also emphasize that for other elements (minerals, vitamins, bioactive proteins), camel meat is comparable to other red meat. The health effect that could be attributed to camel meat consumption, except for low cholesterol which can be a strong commercial argument, is due to the good balance of the different elements that compose it. However, a significant improvement could be expected by better slaughtering and cooking conditions.

The present issue of *Animal Frontiers* cannot cover all the topics regarding camelid science and all disciplines linked to this marginal but emerging species in a context of diversification of livestock farming and growing integration of camel products in a globalized economy. However, it is expected that this special issue will stimulate the scientific interest of animal scientists, especially in Europe, for these strange animals.

*Conflict of interest statement*. None declared.

## About the Authors

**Bernard Faye** (h-index: 34) is a veterinarian who specialized in tropical veterinary medicine. He received his PhD from the Paris University and HDR from the Montpellier University. He stayed in Africa (Ethiopia and Niger) for 8 years (1975 to 1983) for research and development activities before joining the National Institute of Agricultural Research (INRA) as a director of ecopathology laboratory. He joined CIRAD (Centre for International Cooperation in Agricultural Research for Development) as head of the Animal Productions Program (1996) and then as scientific project manager (2007). Starting interest for camels in Ethiopia in 1975, he studied mineral metabolism. Gradually, through his multiple research programs in cooperation and his international network of camel scientists (North-Western and Horn of Africa, Middle-East, India, Central Asia, and Latin America), he founded the International Society for Research and Development on Camelids (ISOCARD). From 2010 to 2015, he worked as a FAO consultant in a camel research center in Saudi Arabia. At present, he is the chairman of ISOCARD and vice-chairman of the French Federation of camelids farming and also an emeritus researcher at CIRAD and an independent international camel expert. He is the author of more than 450 scientific publications, 340 communications in national and international conferences, 45 books and chapters in the books, 210 technical reports, and 12 scientific editions.

Website: http://camelides.cirad.fr and http://www.isocard.net

**Marcelo H. Ratto** is a full professor at the Institute of Animal Science, Faculty of Veterinary Sciences, Universidad Austral de Chile, where he teaches reproductive physiology and biotechnology in undergraduate and graduate courses. He received his DVM from the Universidad Nacional de la Plata, Argentina; an MSc in Animal Reproduction from the Universidad Austral de Chile; and a PhD from the University of Saskatchewan, Canada. He conducts basic and applied research in llamas and ruminants (cows and sheep), thereby investigating the effect of the neurotrophin β-NGF on ovarian and uterine function. He is also interested in incorporating this factor in estrus synchronization protocols for fixed-time artificial insemination in dairy cattle to improve fertility.
